# Exosomal miRNA as biomarker in cancer diagnosis and prognosis: A review

**DOI:** 10.1097/MD.0000000000040082

**Published:** 2024-10-18

**Authors:** Mingliao Zhu, Yuan Gao, Kaijun Zhu, Ying Yuan, Haoyang Bai, Liwei Meng

**Affiliations:** a Medical School of Shaoxing University, Yuecheng, Shaoxing, Zhejiang Province, People’s Republic of China; b Department of Breast and Thyroid Surgery, Shaoxing People’s Hospital, The First Affiliated Hospital of Shaoxing University, Shaoxing, Zhejiang Province, People’s Republic of China.

**Keywords:** biomarker, cancer, diagnosis, exosomal miRNA, prognosis

## Abstract

Exosomes, which are extracellular vesicles with a diameter ranging from 40 to 160 nm, are abundantly present in various body fluids. Exosomal microRNA (ex-miR), due to its exceptional sensitivity and specificity, has garnered significant attention. Notably, ex-miR is consistently detected in almost all bodily fluids, highlighting its potential as a reliable biomarker. This attribute of ex-miR has piqued considerable interest in its application as a diagnostic tool for the early detection, continuous monitoring, and prognosis evaluation of cancer. Given the critical role of exosomes and their cargo in cancer biology, this review explores the intricate processes of exosome biogenesis and uptake, their multifaceted roles in cancer development and progression, and the potential of ex-miRs as biomarkers for tumor diagnosis and prognosis.

## 1. Introduction

Recently, a diagnostic technique known as “liquid biopsy” has garnered significant attention in the field of cancer diagnosis. While tissue biopsy remains the gold standard for diagnosing of tumors, it is not without limitations, including extensive trauma, inconvenient access, and substantial diagnostic errors stemming from tumor heterogeneity.^[1]^ In contrast, liquid biopsy is a noninvasive diagnostic method that can diagnose various diseases, including cancer, by collecting nonsolid biological tissue samples of the human body, such as blood, cerebrospinal fluid, saliva, pleural effusion, ascites, feces, urine, etc.^[2–4]^ It overcomes the problem of tumor heterogeneity and is conducive to the early and accurate diagnosis of tumors.

Exosomes are a kind of extracellular vesicle with a diameter of 40 to 160nm,^[5]^ which play an important role in cell communication and epigenetic regulation by transporting key proteins and genetic materials such as miRNAs in cells.^[6,7]^ Exosomal microRNA (ex-miR) is particularly promising for use in liquid biopsy due to several advantages: (1) ex-miR has high sensitivity and specificity^[8]^; (2) ex-miR is widely present in body fluids, and its properties are highly stable and easily accessible.^[2,9]^ (3) ex-miR is significantly increased in tumors compared with normal tissues. Consequently, ex-miRs hold considerable potential in the early diagnosis of cancer. Studies have demonstrated their significant role in diagnosing various cancers and evaluating cancer prognosis.^[10–20]^

This review initially delves into the biogenesis and uptake mechanisms of exosomes, followed by an exploration of their functions within the context of cancer. Furthermore, it examines the diagnostic utility of exosomes in cancer and their significance in evaluating cancer prognosis. The review clarifies the advantages of ex-miR as a tumor biomarker to stimulate the progress of cancer diagnosis strategies and provide new methods for evaluating cancer prognosis.

## 2. Biogenesis and uptake of exosomes

Exosomes are specialized vesicles for cargo delivery that cells release through the fusion of multivesicular bodies (MVBs) with the plasma membrane.^[21]^ The formation of exosomes is a finely regulated process consisting of 4 steps: MVBs sorting, formation of MVBs, transport of MVBs, and fusion of MVBs with the PM.^[22]^ During MVBs sorting, proteins are labeled by monomer or K63-linked polyubiquitination, which serves as a key signal for their entry into MVBs. ESCRT complex as the key components of the intracellular vesicles transport, by identifying these ubiquitin tag, to choose specificity and separation of the target protein and its orientation MVBs.^[23]^ The orchestration of the ESCRT complex cascade (ESCRT-0, I, II, and III) is pivotal in the biogenesis of MVBs. ESCRT-0 initiates this intricate process by recognizing and aggregating proteins tagged with ubiquitin, thus setting the stage for the genesis of intraluminal vesicles (ILVs). Subsequently, ESCRT-I and ESCRT-II perpetuate the maturation of these ILVs through the intricate process of membrane remodeling. Then, ESCRT-III assumes a helical configuration, which exerts a driving force on membrane budding, culminating in the precise encapsulation of the ILVs. As the number of ILVs within the endosome accumulates progressively, they coalesce to form the MVB structure, encapsulating a multitude of ILVs within their confines.^[24]^ The intracellular trafficking of MVBs is orchestrated by a cohort of Rab GTPases, with Rab7 and Rab27a occupying a pivotal positions. Rab7 exerts primary control over the translocation of MVBs to lysosomes, facilitating the terminal stages of MVB maturation and degradation. Conversely, Rab27a predominantly governs the trafficking of MVBs to the cell membrane, a critical step in the exocytotic pathway that leads to the release of exosomes.^[25]^ Among these steps, the fusion of MVBs with the PM is a complex and vital process, which is a research hotspot. This process is regulated by factors such as molecular switches (small GTPases), the cytoskeleton (microtubules and microfilaments), molecular motors (dynein and kinesin), and membrane fusion devices (SNARE complexes). Rab GTPase is the most critical factor, distributed on the cell membrane surface and regulates MVB budding, motility, and fusion.^[26]^ The primary endosomal GTPase is Rab5. In addition to its role in membrane fusion, Rab5 is also important in the uptake of exosomes. After endocytosis, the Rab5-specific GEF Rabex5 binds to cargo proteins, activates Rab5 on endocytic vesicles, and functions as a fusion of cell membranes by recruiting several effectors on the endosomal membrane like early endosome antigen 1 and the CORVET complex.^[27]^

Studies have demonstrated that the mechanism of MVB fusion mainly includes transport-dependent (ESCRT) and ESCRT-independent.^[22]^ The mechanisms of exosome uptake include endocytosis, direct membrane fusion, and receptor–ligand interaction. In recent years, more and more studies have shown that endocytosis is the primary method for the uptake of exosomes. Exosomes can be internalized via clathrin, lacunin, and lipid raft-mediated endocytosis in different specific receptor cells. In addition, the exosome membrane can fuse with the PM to deliver its contents to the recipient cell or bind to cognate receptors on the recipient cell membrane, subsequently triggering an intracellular signaling cascade.^[26]^

## 3. Exosome functions in cancer

**Figure 1.**
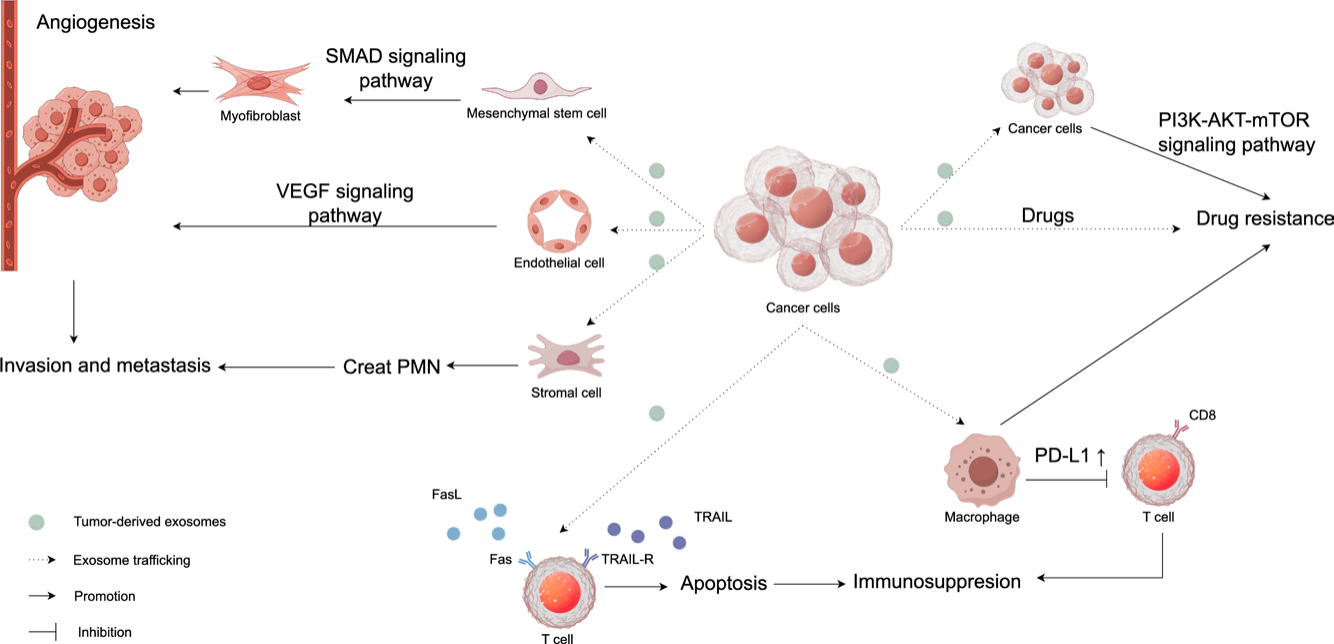
Regulation of the tumor microenvironment by tumor-secreted exosomes (By Figdraw).

The function of exosomes is primarily determined by their contents, including proteins, lipids, and nucleic acids such as DNA, mRNA, and miRNA. Under pathophysiological conditions, changes in the composition and quantity of these components within exosomes can lead to a range of disease-related effects. Exosomes are intricately involved in numerous physiological processes, including cell communication, antigen presentation, immune response, organ development, and reproductive performance.^[28–31]^ Studies comparing normal tissues with tumor tissues have revealed that the exosomes secreted by tumor tissues are 10 times higher in concentration than those secreted by normal tissues.^[32]^ Therefore, the study of exosomes in tumors is potential. Exosomes play a pivotal role in tumor progression and the aggravation of tumorigenesis through a complex interplay of mechanisms. These mechanisms include the promotion of tumor cell metastasis and invasion, the regulation of drug resistance, and the modulation of immune responses, all of which have significant implications for therapeutic outcomes. A schematic figure corresponding to these processes is depicted in Figure 1.

Firstly, exosomes play a pivotal role in mediating tumor metastasis and invasion. The invasive and metastatic capabilities of cancer cells allow them to migrate to distant organs, and exosomes play an essential role in angiogenesis and the creation of pre-metastatic niche.^[33]^ The VEGF signaling pathway is a crucial target for angiogenesis and is important in physiological and pathological angiogenesis. Tumor-derived exosomes can transport a range of angiogenesis-promoting factors, including VEGF, to induce capillary formation.^[34]^ Myofibroblasts are involved in tumor angiogenesis, and it has been reported that tumor-derived exosomes can induce the transformation of adipose-derived mesenchymal stem cells into myofibroblasts through the SMAD pathway.^[35]^ In the tumor microenvironment, tumor cells co-opt stromal cells to create a microenvironment that is conducive to tumor metastasis and progression.^[36]^ It has been confirmed that tumor-derived exosomes are involved in the formation of pre-metastatic niche and promote the distant metastasis of tumors.^[32,37]^

Secondly, exosomes regulate tumor immunity. Cancer progression is closely associated with the dysregulation of immune cells.^[38,39]^ Some tumor tissues secrete exosomes expressing death ligands, which can trigger the death of immune cell. For example, FasL and TRAIL, 2 death ligands secreted by tumor-derived exosomes, can trigger the programmed apoptosis of T cells.^[35]^ Programmed death ligand 1 (PD-L1) has been proven to suppress CD8 T cells. Chen et al showed that human melanoma-derived exosomes carrying the immunosuppressive PD-L1 inhibit CD8 T-cell.^[40]^ Yang et al found that co-culture of Golgi membrane protein 1 with hepatocellular carcinoma (HCC) cell-derived exosomes upregulated PD-L1 expression in macrophages, thereby exacerbating the suppression of HCC CD8 T cells and promoting tumor growth.^[41]^ The dysregulation of immune cells is beneficial to the survival and progression of tumors.

Finally, exosomes regulate tumor drug resistance. In studies of lung, pancreatic, and colorectal cancer, the drug-resistance effect mediated by exosomes has been substantiated.^[42–45]^ There are various mechanisms through which exosomes confer drug resistance in cancer cells. Cancer cells can directly expel drugs from the cells via exosomes.^[34,46]^ Additionally, exosomes can also induce tumor drug resistance by modulating T lymphocytes, macrophages, and other cells.^[47]^ Exosomes contribute to the development of drug resistance in tumor cells by regulating critical signaling pathways such as PI3K–AKT–mTOR, TGF-β, NF-κB, and Bcl-2/Bax.^[48–50]^ Among these pathways, PI3K–AKT–mTOR is a pivotal regulator of cellular growth, proliferation, and metabolism. In cancerous cells, the aberrant activation of PI3K–AKT–mTOR can reduce sensitivity to chemotherapeutic agents, thereby fostering drug resistance. Research has shown that exosomal miR-100-5p targets mTOR, enhancing its expression and consequently augmenting the resistance of tumor cells to chemotherapy.^[51]^ Exosomes derived from cancer stem cells carry miR-210, which, when transferred to pancreatic cancer cells, activates the PI3K–AKT–mTOR signaling pathway and induces resistance to gemcitabine.^[52]^ Tumor-associated macrophages release exosomes containing miR-21, which are delivered to gastric cancer cells. By targeted activation of the PI3K–AKT–mTOR pathway, these exosomes mediate resistance to cisplatin in gastric cancer cells.^[48,53]^

## 4. Exosomal miRNA as biomarker in cancer diagnosis and prognosis

### 4.1. Breast cancer

Regarding the gold standard for diagnosing breast cancer, needle biopsy exhibits high sensitivity and specificity. However, this procedure is invasive. Consequently, finding a diagnostic marker with high sensitivity and specificity and minimum harm to patients is of paramount importance. In current studies, GATA binding protein 3 (GATA3), gross cystic disease fluid protein 15, and mammaglobin are commonly used in diagnosing breast cancer, of which GATA3 is mostly used. Studies have shown that the sensitivities of these 3 markers are low in triple-negative breast cancer, with a range of only 15% to 60%.^[54]^ Recently, the ex-miRs have become a research hotspot. Some studies have found that many exosomes are synthesized and secreted by breast cancer tumor tissues. The concentration of exosomes in peripheral blood is notably higher than that of healthy individuals, leading to a substantial increase in the ex-miR content carried by these exosomes. Therefore, ex-miRs have the potential for early breast cancer diagnosis.^[55]^ Wang et al conducted a study on 55 breast cancer patients and 55 healthy controls and discovered that ex-miR-1910-3p promoted the proliferation and migration of breast cancer cells both in vitro and in vivo by downregulating myotubule-associated protein 3 and activating the NF-κB and wnt/β-catenin signaling pathways. Their research indicated that serum ex-miR-1910-3p is an effective marker for breast cancer diagnosis, with a sensitivity of 88% and a specificity of 76%. And it can improve the sensitivity of breast cancer diagnosis when combined with the traditional tumor marker CA153.^[56]^ Chen et al isolated cancer-associated fibroblasts from breast cancer patients and adjacent normal breast tissue and isolated exosomes using ultracentrifugation. Cancer-associated fibroblasts-derived exosomal microRNAs were screened using next-generation sequencing technology. The studies revealed that exosomal miR-500a-5p was highly expressed in breast cancer cell lines MDA-MB-231 and MCF7. MTT assays and three-dimensioned cultures showed that miR-500a-5p promoted the proliferation of breast cancer cells. However, in vitro transwell assays showed that miR-500a-5p facilitated breast cancer progression and metastasis. Biomarkers serve not only in diagnosing diseases but also in predicting tumor prognosis. Specifically, in breast cancer, emerging biomarkers such as polo-like kinase (PLK) PLK 2, PLK3, and PLK5 have been validated as significant prognostic indicators through survival analysis utilizing the Kaplan–Meier Plotter database. High expression levels of PLK1 and PLK4 have been correlated with poorer recurrence-free survival rates, whereas elevated levels of PLK2, PLK3, and PLK5 indicate better outcomes. This underscores the importance of PLK family members in breast cancer prognosis.^[57]^ Furthermore, in nasopharyngeal cancer studies, biomarkers including ebv-miR-BART19-3p, hsa-miR-135b, and hsa-miR-141 have been confirmed as predictors of survival, providing critical insights for clinical decision-making.^[58]^ Moreover, recent breast cancer research has uncovered the potential of exosomal miRNAs as novel biomarkers, demonstrating their significant promise in improving prognostic assessments.^[59]^ By isolating exosomes from breast cancer tumor tissues and analyzing their expressed miRNAs, Shen et al found that ex-miR-7641 expression levels were significantly increased in the plasma of patients with highly metastatic breast cancer.^[60]^ Yuan et al studied the effect of ex-miR by constructing a mouse model of breast cancer and found that ex-miR-21 promoted bone metastasis of breast cancer cells by regulating PDCD4 protein. They also observed that the expression level of ex-miR-21 was significantly increased in the serum of breast cancer patients with bone metastasis. Consequently, ex-miR-21 may be a potential clinical diagnostic target for bone metastasis of breast cancer.^[61]^ Xun et al developed a suspension co-culture system involving breast cancer cells and macrophages. They determined that ex-miR-138-5p stimulates lung metastasis of breast cancer cells by suppressing the expression of KDM6B in macrophages, and the level of circulating ex-miR-138-5p was found to be positively correlated with the progression of breast cancer.^[62]^

### 4.2. Liver cancer

In recent years, due to the development of imaging technology, significant progress has been made in diagnosing liver cancer. However, due to its insidious nature, most patients are diagnosed at the middle to advanced stages, resulting in poor treatment outcomes and a 5-year survival rate below 20%.^[63]^ In clinical practice, alpha-fetoprotein is a widely used tumor marker for diagnosing liver cancer. However, studies have shown that approximately 50% of early-stage HCC patients are alpha-fetoprotein-negative, making it challenging to diagnose liver cancer accurately and promptly.^[64]^ Similar to breast cancer, needle biopsy serves as the gold standard for diagnosing liver cancer. Nevertheless, its application is limited by its invasiveness, making it less than the ideal diagnostic method. Therefore, it is of great significance to find a biomarker that contributes to the early diagnosis of HCC. Ex-miRs are anticipated to play an essential role in diagnosing liver cancer due to its high sensitivity and specificity.^[65,66]^ Cho et al studied 28 healthy individuals and 90 HCC patients. By comparing the expression levels of ex-miR-215-5p in HCC tumor tissues and normal tissues, they found that the expression level of ex-miR-215-5p was increased by 106.60 times in early HCC. Thus, exo-miR-215-5p holds considerable potential in the diagnosis of early-stage HCC.^[67]^ Suchandrima Ghosh et al extracted 41 ex-miRs from HCC liver cancer tissues. They found that ex-miR-10b, ex-miR-21, and ex-miR-182 were involved in the onset and progression of HCC, among which the sensitivity of ex-miR-21 in distinguishing HCC from non-HCC could reach as high as 87%.^[68]^ In addition, ex-miRs can be utilized to evaluate prognosis of liver cancer. By analyzing the serum ex-miRs of 79 HCC patients, Lee et al found that the expression of ex-MiR-21 was inversely associated with the overall survival of HCC patients, suggesting that ex-MiR-21 can serve as a prognostic biomarker for HCC.^[69]^ Lin et al studied ex-miRs in the body fluid of liver cancer patients associated with tumor metastasis and identified that 119 ex-miRs were upregulated, while 186 ex-miRs were downregulated. Among these, ex-miR-374a-5p was linked to the progression of liver cancer metastasis.^[70]^ These results provide a basis for the development of biomarkers for the diagnosis of liver cancer in the future. Tian et al found that an acidic microenvironment can trigger the activation of HIF-1α and HIF-2α and stimulate the expression of ex-miR-21 and ex-miR-10b, significantly promoting HCC cell proliferation, migration, and invasion. Therefore, measuring the expression of serum ex-miR-21 and ex-miR-10b in HCC patients can be used to evaluate the malignant progression of cancer.^[71]^

### 4.3. Colorectal cancer

Endoscopy combined with pathological biopsy is the primary method for diagnosing colorectal cancer. However, endoscopy is limited due to poor patient tolerance, a high risk of intestinal obstruction, and technical difficulties.^[72]^ Ex-miRs also hold potential for the early and progressive diagnosis of colorectal cancer. By comparing the expression of plasma exosomes between colorectal cancer patients and healthy individuals, Cao et al discovered that the expression level of plasma ex-miR-149-3p was decreased in colorectal cancer patients.^[73]^ Hu et al reported that high expression of ex-miR-92a-3p in serum was highly correlated with the metastasis of colorectal cancer tumors.^[74]^ Sun et al found that ex-miR-135a-5p was associated with liver metastasis, progression, and prognosis of colorectal cancer.^[75]^ Yang et al determined that high ex-miR-106b expression in plasma was associated with the degree of malignant progression of colorectal cancer.^[76]^ Gherman et al selected 31 patients with metastatic colorectal cancer undergoing first-line chemotherapy with irinotecan or oxaliplatin-based regimens. Their findings demonstrated that elevated exosomal expression of miR-92a-3p and miR-221-3p was predictive of a lower overall survival rate in colorectal cancer patients.^[77]^

### 4.4. Lung cancer

Currently, lung cancer detection methods include imaging examination, sputum exfoliative cytology, bronchoscopy, and lung biopsy. However, pulmonary imaging is not conducive to the early detection of lesions due to a high false-negative rate, sputum exfoliative cytology lacks sensitivity, bronchoscopy is associated with numerous complications, and lung biopsy is invasive.^[78]^ Therefore, there is a need to identify a highly sensitive and noninvasive diagnostic tool for lung cancer. Recent studies have shown that the expression of certain ex-miRs is either increased or decreased in lung cancer, indicating the broad prospects of ex-miR as a diagnostic tool. Peng et al analyzed the ex-miR expression profile of non-small cell lung cancer (NSCLC) patients and found that a total of 155 ex-miRs were upregulated.^[79]^ Liang et al studied 68 lung cancer cases and found that ex-miR-144 was under-expressed in lung cancer patients.^[80]^ Wang et al analyzed serum exosomes from lung cancer patients and healthy individuals, discovering that the expression of miR-141 was upregulated in lung cancer serum exosomes.^[81]^ Furthermore, ex-miR, such as ex-miR-23b-3p, ex-miR-10b-5p, and ex-miR-21-5p, are associated with the prognosis, metastasis, and progression of lung cancer.^[82]^ Li et al discovered that the increased ex-miR-10b-5p, ex-miR23b-3p, and ex-miR-21-5p levels and decreased ex-miR-146a-5p levels indicate a poor prognosis for lung cancer.^[42]^ Zhang et al investigated 41 lung cancer patients and found that ex-miR-193a-3p, ex-miR-210-3p, and ex-miR-5100 could be utilized to assess the progression of lung cancer.^[83]^ Yang et al analyzed 30 plasma-derived ex-miRs from NSCLC patients and found that ex-miR-574-5p, ex-miR-328-3p, and ex-miR-423-3p were linked with bone metastasis of lung cancer.^[84]^ Sanchez-Cabrero et al conducted a comprehensive study involving a prospective of 120 samples derived from 88 patients diagnosed with NSCLC. Their findings revealed a notable upregulation of miR-124 concurrent with the advancement of the disease. This observation underscores the potential utility of miR-124 as a prognostic biomarker, specifically in the context of identifying NSCLC patients at an increased risk of recurrence in the early stages of the disease.^[85]^

### 4.5. Others

In addition to breast cancer, liver cancer, colorectal cancer, and lung cancer, the diagnostic role of ex-miR has been reported in various other cancers. For instance, Zheng et al conducted miRNA sequencing on 121 plasma samples from healthy volunteers, cervical cancer patients, and cervical intraepithelial neoplasia patients, ultimately discovering that plasma ex-miR-30d-5p and let-7d-3p were expressed in cervical cancer and its precursors.^[86]^ The findings suggest that ex-miR can be utilized to predict the occurrence of cervical cancer. Zhou, Lanyun et al found that miR-15a-5p, miR-106b-5p, and miR-107 were significantly expressed in exosomes from endometrial cancer patients.^[87]^ Zhu et al observed that the expression level of miR-205 in plasma exosomes was notably increased in ovarian cancer patients with lymph node metastasis.^[88]^ Furthermore, studies have demonstrated that the upregulation of ex-miR-200b and ex-miR-200c is associated with poor survival of ovarian cancer patients.^[89]^ Research in gastric cancer have revealed that ex-miR-139 and ex-miR-513b-5p play an essential role in tumor growth, metastasis, and invasion of gastric cancer cells, thereby serving as a potential target for the diagnosis and treatment of gastric cancer.^[90,91]^ In the study of pancreatic ductal adenocarcinoma, the expression of serum ex-miR-1226-3p in patients with pancreatic ductal adenocarcinoma is downregulated, which can be used as a biomarker for diagnosis and judgment of metastasis or invasion.^[92]^ In addition, ex-miR-4525, ex-miR-451a, and ex-miR-21 have been shown to be associated with the prognosis of pancreatic cancer and can be used to evaluate the recurrence and overall survival of pancreatic cancer.^[93]^ In the investigation of high-grade gliomas (HGG), Olioso et al enrolled 57 newly diagnosed, histologically confirmed HGG patients to validate the utility of serum exosomal miR-21, miR-222, and miR-124-3p as tools for assessing treatment response and early identification of HGG patient progression.^[94]^ Table 1 shows the potential of ex-miRs as biomarkers in different types of cancer.

**Table 1:** Exosomal miRNA in different body fluids and their potential as cancer markers.

References	Cancer type	Exosomal miRNA	Source	Diagnostic/prognostic efficiency
[56]	Breast cancer	miR-1910-3p	Serum	Sensitivity: 88%, specificity: 76%
[62]	Breast cancer	miR-138-5p	Serum	
[60]	Breast cancer	miR-7641	Plasma	
[61]	Breast cancer	miR-21	Serum	
[67]	Liver cancer	miR-215-5p	Serum	The level of ex-mR-215-5p is increased by 106.60 times
[42]	Liver cancer	miR-10b-5p, miR-221-3p, miR-21-5p, miR223-3p	Tissue, blood	miR-10b-5p, sensitivity: 76%, sensitivity: 55%, accuracy: 63%; miR-221-3p, sensitivity: 87%, sensitivity: 52%, accuracy: 65%; miR-21-5p, sensitivity: 74%, sensitivity: 77%, accuracy: 76%; miR223-3p, sensitivity: 61%, sensitivity: 70%, accuracy: 66%.
[69]	Liver cancer	miR-21	Serum	
[70]	Liver cancer	miR-374a-5p	Cell supernatant	
[71]	Liver cancer	miR-21, miR-10b	Serum	
[73]	Colorectal cancer	miR-149-3p	Plasma, feces	
[74]	Colorectal cancer	miR-92a-3p	Serum	
[75]	Colorectal cancer	miR-135a-5p	Serum, cell culture	
[76]	Colorectal cancer	miR-106b	Plasma	
[77]	Colorectal cancer	miR-92a-3p, miR-221-3p	Plasma	Low expression of miR-143-3p and miR-146a-5p, in conjunction with high expression of miR-92a-3p and miR-221-3p, predicts a favorable response to chemotherapy.
[78]	Lung cancer	miR-320d, miR-320c, miR-320b, miR-125b-5p	Plasma	
[79]	Lung cancer	miR-144	Tissue	
[80]	Lung cancer	miR-141	Serum	
[83]	Lung cancer	miR-193a-3p, miR-210-3p, miR-5100	Bone marrow aspirates, plasma	
[84]	Lung cancer	miR-574-5p, miR-328-3p, miR-423-3p	Plasma	
[85]	Lung cancer	miR-124	Tissue and blood samples	Elevated serum levels of miR-124 correlate with increased recurrence and mortality risks in early-stage NSCLC patients, suggesting a poorer prognosis.
[86]	Cervical cancer	Let-7d-3p, miR-30d-5p	Plasma	
[87]	Endometrial cancer	miR-15a-5p, miR-106b-5p, miR-107	Plasma	The AUC for each exo-miR ranged from 0.693 to 0.819 with a mean of 0.757. The AUC of the combined 6 exo-miRs achieved 0.983.
[88]	Ovarian cancer	miR-205	Plasma	The AUC of plasma exosomal miR-205 was 0.715 (95% CI: 0.590–0.841, *P* = .002), with a sensitivity of 66.7% and a specificity of 78.1%.
[90]	Gastric cancer	miR-139	Plasma and tumor samples	
[91]	Gastric cancer	miR-513b-5p	Cell culture	
[92]	Pancreatic cancer	miR-1226-3p	Serum	AUC: 0.74
[94]	High-grade gliomas	miR-21, miR-222, and miR-124-3p	Serum	The upregulation of miR-21, miR-222, and miR-124-3p correlates with the progression of HGG.

## 5. Summary and prospect

The clinical diagnostic imaging techniques for most cancers often fail to provide early and accurate diagnosis. The gold standard for diagnosis, which is invasive detection, may cause harm to patients. Therefore, there is an urgent need for a noninvasive and efficient detection method. Ex-miR, as potential tumor biomarker, exhibit high sensitivity and specificity in the early diagnosis of cancer, assessment of cancer progression, and evaluation of cancer prognosis. This reviews aims to explore the role of ex-miRs in the diagnosis and prognosis of tumors, providing new perspectives for the early diagnosis and prognosis evaluation of tumors.

However, exosomes also have limitations as tumor biomarkers. The most commonly used method to isolate exosomes is the ultracentrifuge.^[95]^ The complexity in their isolation and analysis arises from the fact that exosomes can be secreted by various cell types and carry intricate cargoes, including DNA, RNA, and proteins.^[96,97]^ Moreover, while research has indicated that certain exosomes exhibit high sensitivity and specificity as biomarkers for malignancies, their clinical utility as biomarkers necessitates further substantiation through large-scale clinical studies.^[97]^ Additionally, the lack of a standardized method for the isolation and characterization of exosomes impedes the reproducibility and scalability of research, despite the availability of various techniques, such as ultracentrifugation and size exclusion chromatography, each with its own merits.^[95,98]^ Furthermore, the high costs and technical sophistication required for exosome analysis can lead to substantial financial burdens for research and clinical application, potentially limiting their widespread adoption.^[99–101]^ Nonetheless, it is clear from this review that the potential of ex-miRs as biomarker is undeniable. We have discussed the advantages of liquid biopsy compared with traditional tissue biopsy and listed the diagnostic and prognostic roles of ex-miRs as biomarkers in breast cancer, liver cancer, colorectal cancer, lung cancer, and other cancers.

## Author contributions

**Conceptualization:** Yuan Gao, Liwei Meng.

**Funding acquisition:** Liwei Meng.

**Methodology:** Mingliao Zhu, Yuan Gao.

**Resources:** Kaijun Zhu, Ying Yuan, Haoyang Bai.

**Supervision:** Yuan Gao, Liwei Meng.

**Validation:** Mingliao Zhu.

**Writing – original draft:** Mingliao Zhu.

**Writing – review & editing:** Mingliao Zhu.
